# Risk indicators of aggressive periodontitis in a Jordanian population

**DOI:** 10.1186/s12903-019-0826-1

**Published:** 2019-07-16

**Authors:** Khansa T. Ababneh, Manal J. Maslamani, Muna S. Abbadi, Anas H. Taha, Jumana A. Karasneh, Amani G. Sa’di, Yousef S. Khader

**Affiliations:** 1 Department of Preventive Dental Science, College of Dentistry, King Saud bin Abdulaziz University for Health Sciences, Riyadh, Kingdom of Saudi Arabia; 2 Previous Department of Preventive Dentistry, Faculty of Dentistry, Jordan University of Science and Technology, Irbid, Jordan; 3 Restorative Department, Kuwait University, Kuwait City, Kuwait; 4 Private practice, Amman, Jordan; 5 Private practice, Abu Dhabi, United Arab Emirates; 6 Oral Medicine, and Human Molecular Genetics, Department of Oral surgery, Oral Medicine, Oral Pathology and Radiology, Faculty of Dentistry, and Faculty of Science and Art, Biotechnology and Genetic Engineering Department, Jordan University of Science and Technology, Irbid, Jordan; 7 Jordanian Food and Drug administration, Amman, Jordan; 8 Community Medicine & Public Health, Department of Public Health and Community Medicine, Faculty of Medicine, Jordan University of science and Technology, Irbid, Jordan

**Keywords:** Aggressive periodontitis, Risk indicators, Jordan

## Abstract

**Background:**

Knowledge of the risk indicators of aggressive periodontitis (AgP) will help clinicians to better diagnose the disease, put a treatment plan that involves modification of modifiable risk indicators, understand non-modifiable risk indicators, and may potentially serve as an aid in developing preventive programs. The objective of the present study was to assess risk indicators of aggressive periodontitis (AgP) in Jordan including socio-demographic factors, oral hygiene habits, smoking, family history and parents’ consanguinity.

**Methods:**

A total of 162 patients (81 AgP and 81 controls), attending the Periodontology clinic at Jordan University of Science and Technology, Dental Teaching Centre, were interviewed and examined. All AgP subjects had full periodontal and radiographic examination. The data recorded included socio-demographic and economic variables, oral hygiene and smoking habits, family history and parents’ consanguinity.

**Results:**

Most AgP patients were young females, had ≤12 years of education, lived in urban areas and brushed their teeth ≥ once daily. Risk indicators of AgP included: age > 35 years, female gender and positive family history.

**Conclusions:**

Risk indicators associated with AgP in this study population were: age > 35 years, female gender and positive family history of periodontal disease.

## Background

Periodontitis is a multifactorial disease characterized by attachment and bone loss around teeth. The incidence and progression of the disease are influenced by the interaction of genetic, microbial and environmental factors such as dental plaque (biofilm), age, gender, ethnicity, systemic diseases, smoking, psychological, genetic polymorphisms and social factors [1–3]. The American Academy of Periodontology has classified periodontitis into chronic periodontitis (CP), aggressive periodontitis (AgP), and periodontitis associated with systemic diseases [4]. Case definition of AgP has been modified several times, from the early work of Orban and Weinmann (1942) [5], who introduced the term “periodontosis”, through the more elaborate definition presented by Baer in (1971) [6], to the latest case definition proposed by the AAP, in 1999. [4]. The 1999 AAP report states that AgP affects otherwise healthy individuals and is characterized by rapid attachment and bone destruction at an early age, familial aggregation, phagocyte abnormalities, the presence of a hyper-responsive macrophage phenotype, elevated levels of prostaglandin E2 and interleukin-1ß, and alteration in the host immune response [4, 7].

A systematic review of literature by Susin et al., (2014) on the epidemiology and demographics of AgP [8], has demonstrated that the prevalence of AgP in Europe ranges between 0.1–0.2%; in North America 0.5–1.7%, depending on the case definition and sample type (age, ethnicity); in South America from 0.3–3.7%; In Asia varied form 1.8–5.9% and in Africa from 0.7–8.6%. This review has highlighted two main facts; the first is that AgP is a significant health problem in certain populations and the second is the lack of information on the epidemiology of AgP in many parts of the world. Epidemiological studies of AgP in high-risk populations are important and could provide vital data on the determinants of this disease, and this information is needed for the establishment of effective health-promotion measures [8].

The risk indicators examined in this study included demographic and socioeconomic factors, oral hygiene habits, smoking, family history and parents’ consanguinity. The reason for selecting these specific risk indicators is the strong association between them and periodontitis, as demonstrated by other researchers [9].

## Patients and methods

### Study design

The present study is a case-control study, which involved 162 patients attending Jordan University of Science and Technology (JUST) Dental Teaching Center (DTC) in Irbid. The subjects included were ≥ 14 years of age and denied having any medical problems. The disease group consisted of 81 AgP patients and the control group consisted of 81 periodontitis-free subjects.

### Exclusion criteria

Patients who reported any medical or physiologic condition associated with periodontitis and listed in the latest AAP classification [4], including diabetes mellitus, blood disorders, immunosuppression, pregnancy, patients on long term medications such as contraceptives and steroids were excluded from the study. Subjects who had received periodontal treatment in the last 3 months prior to examination, and patients with current or previous orthodontic treatment were also excluded.

### Interview, clinical and radiographic examination

This study was conducted in full accordance with ethical principles of the World Medical Association Declaration of Helsinki [10].

Approval for this study was obtained from the Ethical Committee in the Deanship of Scientific Research, IRB, JUST, and informed consent forms for interview and examination were signed by all subjects and the parents of subjects under the age of 18 years. For each subject, a questionnaire was completed and included: age, gender, occupation, education level, presence of periodontal problems among other family members, parents’ consanguinity, smoking habits, and oral hygiene habits.

Full mouth periodontal examination was carried out which included measurement of Clinical Attachment Level (CAL), the gingival index (GI) of Löe and Silness [11] and the plaque index (PI) of Silness and Löe [12]. For measurement of CAL, each tooth was examined by “walking” the periodontal probe around the whole circumference of the tooth. CAL was measured at six sites per tooth (mesio-, mid-, and disto-buccal; mesio-, mid-, and disto-lingual/palatal). Third molars and remaining roots were excluded. Inter-examiner reliability regarding probing depth and CAL was calculated on 16 quadrants, using alpha statistics. Diagnosis of AgP was based on CAL values and confirmed radiographically using intra-oral periapical and bitewing radiographs. For all participants bitewing radiographs were taken for posterior teeth and periapical radiographs were taken for anterior teeth to detect the presence and pattern of alveolar bone loss and confirm (or exclude) the presence of periodontitis. AgP was diagnosed when the subject had CAL ≥ 3 mm around at least two teeth, one of which was a first molar, or when attachment loss was observed around first molars and/or incisors that exhibited bone loss, especially were the characteristic arc-shaped defect(s) was/were detectable on radiographs, the case was diagnosed as AgP. Inconsistence between the amount of plaque deposits and the amount of periodontal destruction (whenever present), and positive family history further confirmed the diagnosis of AgP. Cases where there was any uncertainty between the diagnosis of AgP or CP were not included in this study.

### Controls

The control sample consisted of 81 periodontitis-free Jordanian subjects, none of which demonstrated attachment or bone loss at any site. The control subjects received full periodontal examination to confirm that they were periodontitis-free.

### Statistical analysis

Data were entered into a personal computer and analyzed using the Statistical Package for Social Sciences (SPSS) software version 11.0 (SPSS®: Inc., Chicago, IL, USA). Frequency distribution, means and standard deviations were calculated. Independent t- and chi square tests were used for comparison among groups as appropriate. Furthermore, multivariate logistic regression model analyses, analyzing the association between explanatory (independent) and dependant variables, were performed to test the association of the outcomes with the independent variables that were included in the models by using backward stepwise Wald method (BSTEP). In this BSTEP method, all the possible variables were entered into the model. The independent variables specified in the variables list were then tested for possible removal from the model one by one at each step, based on the level of significance in the Wald statistics. The variable with the smallest significance composed to PIN (probability for entry) (0.05) was left in the model. If the significance level was greater than POUT (probability for removal) (0.1) the variable was removed. The algorithm stopped when no more variables could be entered or removed. Adjusted odds ratios (OR) were generated corresponding to 95% confidence intervals (CI) for all significant variables. The level of significance was set at (*P* ≤ 0.05).

## Results

### Socio-demographic characteristics

The study population was divided into two age groups (Table 1). The age of the AgP group ranged between 16-46 years with a mean of 29.7 years; whereas the age of the healthy group ranged between 14-37 years with a mean of 22.3 years. Although the majority of AgP patients and controls were ≤ 35 years of age, there was a statistically significant difference between the two groups (*P* = < 0.0001). The majority of AgP patients were females (Table 1), which represented a significant difference between AgP and controls. As for education, the majority of AgP patients have studied up to high school (≤12 years), whereas the majority of controls were highly educated (> 12 years), with a statistically significant difference. The majority of AgP patients and controls reported living in urban areas, but there was no significant difference between the periodontitis and control groups.

**Table 1:** Socio-demographic Characteristics of the Study Population

Variable	AgP N (%) *N* = 81	Control N (%) *N* = 81	Significance* AgP vs. control
Age			< 0.0001
≤ 35	56 (69.1)	76 (93.8)	
> 35	25 (30.9)	5 (6.2)	
Age Mean (Yrs)	29.75	22.3	< 0.0001
Gender			< 0.0001
Male Female	24 (29.6)	46 (56.8)	
	57 (70.4)	35 (43.2)	
Education			0.007
≤ 12	42 (51.9)	25 (30.9)	
> 12	39 (48.1)	56 (69.1)	
Residency			0.081
Rural	28 (34.6)	18 (22.2)	
Urban	53 (65.4)	63 (77.8)	

*χ^2^ test

### Oral hygiene habits and frequency of dental visits

Table 2 shows that the majority of subjects in the disease and control groups reported brushing their teeth ≥1 time/day, but the difference between them was not significant. The highest percentage of AgP subjects reported using a vertical (scrub) method, whereas the highest percentage of controls reported using a simple circular method. The majority of all subjects reported attending dental clinics for emergency treatment only. About 22% of AgP cases and 8.5% of controls reported attending dental clinics regularly.

**Table 2:** Oral Hygiene Habits and Pattern of Dental Visits

Variable	AgP N (%) N = 81	Control N (%) N = 81	Significance* AgP vs. control
Tooth brushing frequency			0.072
< 1	20 (24.7)	11 (13.6)	
≥ 1	61 (75.3)	70 (86.4)	
Method of brushing			0.007
Horizontal	10 (12.5)	8 (10.4)	
Vertical	35 (43.8)	18 (23.4)	
Circular	7 (8.8)	21 (27.3)	
Stillmans	7 (8.8)	14 (18.2)	
H + V	14 (17.5)	7 (9.1)	
Others	7 (8.8)	9 (11.7)	
Dental visits			0.558
On emergency	63 (77.8)	66 (81.5)	
Regular	18 (22.2)	15 (18.5)	

*χ^2^ test

### Cigarette smoking, family history and parent consanguinity

Table 3 shows that about 20% of the whole study population were cigarette smokers. This Table demonstrates that 16% of AgP patients and 17.3% of controls reported smoking. All AgP smoker patients were light smokers (< 10 cigarettes/day), and have smoked for > 5 years. In the control group, 71.4% reported smoking ≥10 cigarettes and about 43% reported smoking for > 5 years. There was a significant difference between AgP patients and controls regarding the number of smoked cigarettes/day (P = < 0.0001). Table 3 also demonstrates that positive family history of periodontal disease was more frequently reported by AgP patients than controls, with a statistically significant difference.

**Table 3:** Smoking, Family History and Parents’ Consanguinity

Variable	AgP N (%) N = 81	Control N (%) N = 81	Significance* AgP vs. control
Smoking			0.833
No	68 (84.0)	67 (82.7)	
Yes	13 (16.0)	14 (17.3)	
Number of cigarettes/day			< 0.0001
< 10	13 (100.0)	4 (28.6)	
≥ 10	0 (0.0)	10 (71.4)	
Duration of smoking (yr)			0.332
< 5	5 (38.5)	8 (57.1)	
≥ 5	8 (61.5)	6 (42.9)	
Family history			0.012
No	34 (42.0)	50 (61.7)	
Yes	47 (58.0)	31 (38.3)	
Parents’ consanguinity			0.335
No	46 (56.8)	52 (64.2)	
Yes	35 (43.2)	29 (35.8)	

*χ^2^ test

### Periodontal parameters

Table 4 shows that the AgP group had significantly higher GI, PI and CAL values than the control group.

**Table 4:** Clinical Parameters of the Study Population

Variable	AgP Mean ± SD N = 81	Control Mean ± SD N = 81	Significance* AgP vs. control
GI	1.69 ± 0.64	1.086 ± 0.707	< 0.0001
PI	1.5 ± 0.76	1.063 ± 0.753	< 0.0001
CAL (mm)	2.76 ± 1.77	00.00 ± 00.00	< 0.0001

*Independent t-test

### Multivariate analysis

Table 5 shows the risk indicators contributing to AgP. Backward stepwise multiple logistic regression identified the following risk indicators for AgP compared to controls: age > 35 years, female gender and positive family history. Subjects age > 35 years was the strongest indicator associated with AgP (OR = 10.12). When patients were older than 35 years they were about ten times more likely to have AgP compared with the younger group (≤35 years). When the patients were female, they were about four times more likely to have AgP in comparison with males. In addition, patients who reported positive family history of periodontitis were twice more likely to have AgP compared with those who reported negative history.

**Table 5:** Multiple logistic regression model for AgP and CP (*N* = 262)

Dependent variable	Independent variable	Significance	Odds Ratio	95% CI-OR
AgP vs. control	Age (> 35 Yrs)	< 0.0001	10.12	3.3-30.9
	Gender (Female)	< 0.0001	3.88	1.86-8.13
	Family history (+ve)	0.037	2.11	1.05-4.24

## Discussion

Studying risk indicators of periodontitis provides dental clinicians with insight into the causative and possible contributing factors of this unique and complex disease in their society. This would improve diagnosis, treatment planning, treatment, prevention and referral of AgP cases.

### Socio-demographic factors

#### Age

The majority of AgP patients were young, which is in agreement with the tendency of AgP to start early in life [13]. Albandar et al. [14] reported that the prevalence of EOP in Ugandan school attendees aged 12-25 years was high (28.8%). The 1999 AAP classification has minimized the value of age in the diagnosis of AgP, although still stating that AgP affects young individuals [7]. Interestingly, the multivariate analysis showed that the odds of having AgP in subjects older than 35 yrs. was 10 times higher than in those under 35 yrs. This result was probably obtained due to two factors: first; the control subjects were younger than AgP subjects, simply meaning that they were young and didn’t have periodontitis. Second; the diagnosis of periodontitis was based on CAL values, so that this “higher odds of having AgP with increasing age” probably reflects the cumulative effect of AgP (manifested as greater attachment loss) that had affected these patients at a younger age, and progressed with increasing age. This finding is supported by the results of other studies [8, 15]. Such as the study of Albandar et al. [8] who estimated the prevalence of aggressive periodontitis in US schoolchildren to be 0.4% among 13- to 15-year old children and 0.8% among the 16- to 19-year-old group. Another study investigated 13-year-old Brazilian children at baseline and 3 years later and found a higher percentage of AgP in the older age group [7, 16].

#### Gender

More than one half (57%) of the participants in this study were females. Females represented most of the AgP group, as opposed to controls, where the number of males was higher. The multivariate logistic regression analysis revealed that females were about four times more likely to have AgP, in agreement with some studies on Caucasians [8, 17].

A recent comprehensive review of the literature on the prevalence and demographics of AgP by Susin et al. [15] showed that there is a complex relationship between the prevalence of AgP, gender, and certain demographic variables, such as race/ethnicity. This review showed that in most populations the prevalence of aggressive periodontitis is similar in male and female subjects. A survey involving 17-26 yrs. old American recruits [18], found that the prevalence of juvenile periodontitis was similar in males and females. However, they observed a significantly higher prevalence of juvenile periodontitis in males than in females when only black recruits were studied, indicating that gender distribution of AgP differs between ethnic groups [18]. This study reported the following female: male ratios of disease prevalence: 0.52:1 in Black people; 4.3:1 in Caucasians; and 3:1 in other races.

### Education and residency

The majority of AgP patients had received ≤12 years of education (equal to or less than high school). Previous studies have reported that education and place of residence are important factors in periodontal health but education has a greater influence on the level of periodontitis [19, 20]. Most AgP patients in our study reported living in urban areas, which may indicate that AgP patients living in the city seek periodontal treatment more frequently than rural area dwellers. Rural areas often have poorer socioeconomic conditions and medical facilities than urban areas.

### Oral hygiene habits, dental visits and periodontal parameters

Dental plaque is the principal etiologic factor of periodontitis as demonstrated by the early studies of Löe and co-workers [21]. In the present study, most AgP patients reported brushing their teeth rather regularly (≥ 1 time/day). But in spite of that, most of them had dental plaque and gingival inflammation. This may either indicate that the tooth brushing methods used by patients were incorrect or that their reports were inaccurate. Controls reported adequate brushing frequency, proper tooth brushing methods and demonstrated better periodontal conditions than AgP patients. This is probably because most of the control subjects were young educated individuals who took care of their oral health. Axelsson et al. [22] reported that intensive oral hygiene programs were effective in reducing the incidence of dental caries and the level of gingival inflammation in children and adults. As for the frequency of dental visits, AgP patients visited the dentist more frequently than controls, possibly due to their need for continuous periodontal treatment and/or replacement of missing teeth.

With regard to the presence of local etiologic factors, although the oral hygiene habits reported by the AgP and control groups were comparable, the AgP patients demonstrated higher plaque scores and attachment loss. AgP has been associated in the literature with minimal amounts of plaque [13], which is in contrast with findings in our AgP population, who demonstrated more plaque than controls. This is probably related to differences between populations in oral hygiene standard and dental awareness. The attachment loss observed in the AgP group probably reflects periodontal response to dental plaque and the high susceptibility of AgP patients to periodontal breakdown. Classically, AgP has been associated with small amounts of local factors [7]. Periodontal destruction in AgP is initiated by the interaction between pathogenic microorganisms and the host immune system [23, 24], with a pronounced role of the host immune reactions (reviewed by Albandar, 2014) [7].

### Smoking

Evidence suggests a very strong association between smoking and gingival tissue status, periodontal tissue loss and severity of periodontitis [3, 25]. In the present study, the percentage of smokers was 16.7% of the whole study population. The majority of subjects in the AgP group denied smoking. This indicates that AgP patients were not heavily exposed to smoking; nevertheless, they had greater periodontal destruction, which supports the high susceptibility of AgP patients to periodontal breakdown. The reason why the percentage of AgP smoker patients was not high may be that some AgP patients could have become aware of the risks of smoking during their visits to dental professionals. Alternatively, this may be due to the fact that the majority of AgP patients were young females who usually deny smoking in the Jordanian society, as it is considered inappropriate for females to smoke.

Although smoking is a well-known and universally accepted risk factor for the initiation, progression and severity of periodontitis [26], the results of the multivariate analysis in this study did not reveal any association between smoking cigarettes, cigarette numbers or duration of smoking and periodontal status. This disagreement may be due to variations among studied populations, sample number and, mostly, accuracy and subjectivity of self-reporting by patients.

### Family history and parents’ consanguinity

It is well recognized that AgP aggregates in families [7, 15, 27]. A currently widely held view is that the destruction observed in periodontal disease is the result of an improperly regulated immune response to bacterial infection rather than the directly destructive effect of the bacterial pathogens themselves [28]. Specific genotypes, such as polymorphisms in IL-1 [29, 30] genes have been linked to increased risk of periodontal diseases [31].

In the present study, more than half of AgP patients reported periodontal problems among other family members, and by that, significantly differed from controls, in agreement with other studies [32]. Familial aggregation of periodontitis may result from shared genes, shared environmental exposures and similar socioeconomic influences. Marriage between relatives, particularly cousins, as a social habit in the Jordanian society was not found to be a risk indicator of AgP in the current study. This is in agreement with previous results [33, 34], but additional studies are recommended to investigate this factor. Marriage between relatives can be looked at as “inbreeding”, the impact of which is well documented on Mendelian disorders. However, very little is known on the effects of inbreeding on late onset and complex, multifactorial diseases such as periodontitis. A study conducted on an Israeli-Arab community reported that in spite of the high rate of consanguinity, no significant difference was found in the prevalence of complex diseases like diabetes, myocardial infarction and asthma between the offspring of consanguineous versus non-consanguineous parents [34].

## Conclusions

The followings risk indicators were associated significantly with AgP in comparison with controls: age > 35 years (indicating the cumulative effect of AgP), female gender and positive family history of periodontal problems. With respect to education, AgP patients have studied for a significantly lower number of years that control subjects, which emphasizes the role of education in prevention of periodontal diseases. AgP patients had significantly greater PI, GI, and periodontal destruction than controls. The Oral hygiene of the study population was poor, which points to a great need for construction of nationwide educational and preventive programs.

## Data Availability

The datasets generated during the current study are available upon reasonable request.
